# The Safety, Effectiveness and Concentrations of Adjusted Lopinavir/Ritonavir in HIV-Infected Adults on Rifampicin-Based Antitubercular Therapy

**DOI:** 10.1371/journal.pone.0032173

**Published:** 2012-03-07

**Authors:** Eric H. Decloedt, Gary Maartens, Peter Smith, Concepta Merry, Funeka Bango, Helen McIlleron

**Affiliations:** 1 Division of Clinical Pharmacology, Department of Medicine, Groote Schuur Hospital, University of Cape Town, Cape Town, South Africa; 2 Department of Pharmacology, St James's Hospital, Trinity College Dublin, Dublin, Ireland; 3 Ubuntu Clinic, Site B Khayelitsha Day Hospital, Khayelitsha, Western Cape Department of Health, Cape Town, South Africa; University of New South Wales, Australia

## Abstract

**Objective:**

Rifampicin co-administration dramatically reduces plasma lopinavir concentrations. Studies in healthy volunteers and HIV-infected patients showed that doubling the dose of lopinavir/ritonavir (LPV/r) or adding additional ritonavir offsets this interaction. However, high rates of hepatotoxicity were observed in healthy volunteers. We evaluated the safety, effectiveness and pre-dose concentrations of adjusted doses of LPV/r in HIV infected adults treated with rifampicin-based tuberculosis treatment.

**Methods:**

Adult patients on a LPV/r-based antiretroviral regimen and rifampicin-based tuberculosis therapy were enrolled. Doubled doses of LPV/r or an additional 300 mg of ritonavir were used to overcome the inducing effect of rifampicin. Steady-state lopinavir pre-dose concentrations were evaluated every second month.

**Results:**

18 patients were enrolled with a total of 79 patient months of observation. 11/18 patients were followed up until tuberculosis treatment completion. During tuberculosis treatment, the median (IQR) pre-dose lopinavir concentration was 6.8 (1.1–9.2) mg/L and 36/47 (77%) were above the recommended trough concentration of 1 mg/L. Treatment was generally well tolerated with no grade 3 or 4 toxicity: 8 patients developed grade 1 or 2 transaminase elevation, 1 patient defaulted additional ritonavir due to nausea and 1 patient developed diarrhea requiring dose reduction. Viral loads after tuberculosis treatment were available for 11 patients and 10 were undetectable.

**Conclusion:**

Once established on treatment, adjusted doses of LPV/r co-administered with rifampicin-based tuberculosis treatment were tolerated and LPV pre-dose concentrations were adequate.

## Introduction

In resource constrained settings the second-line antiretroviral therapy (ART) regimen is based on ritonavir-boosted protease inhibitors (PIs), usually co-formulated lopinavir/ritonavir (LPV/r). Although ART reduces the risk of tuberculosis, incident cases continue to occur on ART at rates higher than the general population [1]. Rifampicin potently induces cytochrome (CYP) 3A4 and p-glycoprotein, resulting in more than a 90% reduction in LPV concentrations [2]. Doubling the dose of LPV/r or adding additional ritonavir (so that LPV∶ritonavir = 1∶1) can overcome the inducing effect of rifampicin [3], [4]. Adjusting doses of PIs to overcome induction by rifampicin resulted in very high rates of hepatotoxicity in healthy volunteers [5]–[7], but we have demonstrated that doubling the dose of LPV/r is relatively safe amongst HIV-infected patients established on LPV/r-based ART [4]. The safety and efficacy of adjusted dose LPV/r in HIV-infected patients with tuberculosis is unclear. Standard tuberculosis treatment includes isoniazid [8], [9], which inhibits CYP 3A4 and may attenuate the inducing effect of rifampicin on lopinavir metabolism. Toxicity may also be different in patients receiving combination tuberculosis treatment. We prospectively followed-up patients on adjusted doses of LPV/r-based ART regimens who were treated with rifampicin-based regimens for tuberculosis.

## Methods

We prospectively enrolled HIV-infected adults older than 18 years from antiretroviral clinics in Cape Town, South Africa, who were on concomitant treatment with rifampicin-based tuberculosis treatment and a LPV/r-based ART regimen. The LPV/r-based ART regimen forms part of second-line ART as recommended by the WHO for developing countries. Doses of LPV/r were adjusted in a non-randomised fashion by the treating clinicians as per national guidelines when tuberculosis treatment was initiated: either doubling the dose of the tablet formulation of LPV/r (800 mg/200 mg 12 hourly) or adding additional ritonavir (LPV/r 400 mg/100 mg plus ritonavir 300 mg 12 hourly). The dosing approach choice was left to the treating clinician. All formulations used were from the originator pharmaceutical company, Abbott. Patients were followed up monthly until 1 month after tuberculosis treatment completion. During each study visit we measured alanine transaminase (ALT). Treatment adherence was assessed using a 3-day treatment recall questionnaire. Patients were asked about the timing of their last LPV/r dose. All adverse events were recorded and graded according to the grading system of the Division of AIDS [10]. Lopinavir pre-dose concentrations were measured every second month and were available within 2 weeks of sampling in order to allow dose adjustment at the discretion of the attending clinician. On the last study visit we measured the viral load.

Plasma lopinavir concentrations were assayed as previously described using liquid chromatography tandem mass spectrometry [11]. The assay range for lopinavir was 0.05–20 µg/ml. Inter- and intra-day coefficients of variation were below 10%. The laboratory participates in the International Interlaboratory Control Program of Stichting Kwaliteitsbewaking Klinische Geneesmiddelanalyse en Toxicologie (KKGT; Hague, The Netherlands). Lopinavir concentrations reported as below the limit of quantification were assigned a value of 0.025 µg/ml. We accounted for repeated measures by calculating the mean lopinavir concentration in each patient. We used the individual mean lopinavir concentrations to calculate the median lopinavir concentration for each dosing group. The HIV viral load was measured using the Abbot HIV viral load assay with a lower limit of quantification of 40 copies per ml. The study was approved by the University of Cape Town Human Research Ethics Committee. Written informed consent was obtained from every participant. The study was registered on the Panafrican Clinical Trials registry (http://www.pactr.org) and the South African National Clinical Trail Registry (http://www.sanctr.gov.za) registry number DOH-27-1108-2594.

## Results

**Figure 1:**
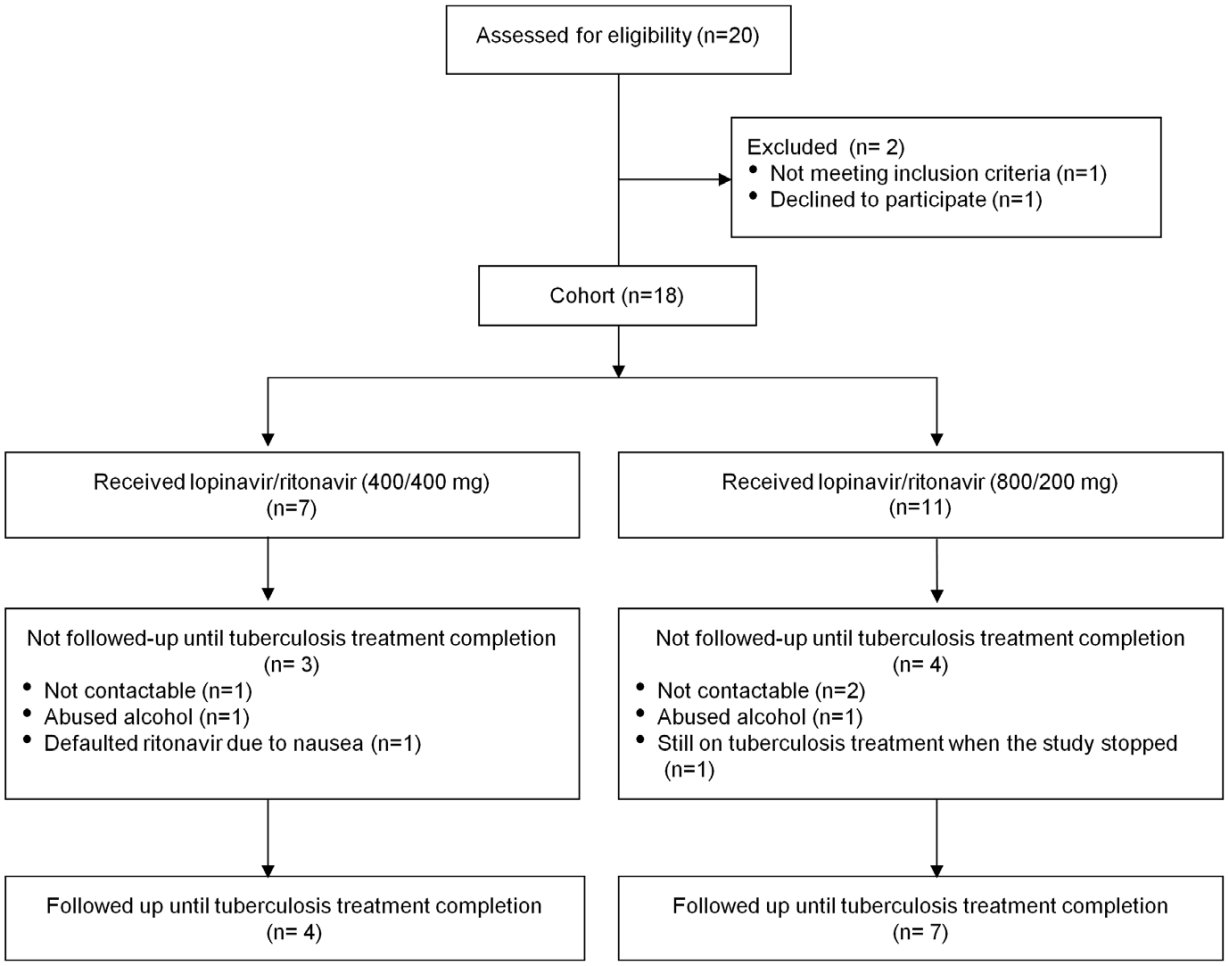
The profile of the study cohort.

We enrolled 18 treatment experienced patients, of whom 11 were female (Figure 1). Eleven patients received double dose LPV/r and 7 received additional ritonavir. One patient was changed from doubled doses of LPV/r to standard doses of LPV/r plus additional ritonavir because of different dosing practices at the health care facility where the patient was transferred. One patient was HBsAg positive. Tuberculosis treatment was dosed according to weight: 12 patients received 600 mg, 5 received 450 mg and the dose was not recorded for 1 patient. In the cohort, the median (IQR) age was 38.5 (33–47) yrs and the median (IQR) CD4-count was 111 (41–181) cells/mm^3^ (Table 1). The median (IQR) month on tuberculosis treatment when enrolled was 4 (2–5) with a total of 79 patient months of observation. Eleven patients were followed up until after tuberculosis treatment completion, 6 were lost to follow-up (3 in each dosing group) and 1 was still receiving tuberculosis treatment when the study was ended. Of the 6 patients that were lost to follow-up; 3 were not contactable, 2 abused alcohol and in one health care staff identified adherence difficulties. In the 7 patients that were not followed up until tuberculosis treatment completion, the median (IQR) time to tuberculosis treatment completion was 1 (0–3.5) months.

**Table 1:** The baseline characteristics of the enrolled cohort.

	Double dose LPV/r (800 mg/200 mg) 12 hourly(n = 11)	Additional ritonavir(400 mg/400 mg) hourly(n = 7)	Total(n = 18)
WeightMedian (IQR)	56 (53.5–59) kg	59.1 (61.2–70) kg	57 (54–63) kg
CD4-count^1^Median (IQR)	80 (37–424) cells/mm^3^	145 (88–167) cells/mm^3^	111(41–181) cells/mm^3^
Viral load^2^	<40 copies per ml: n = 3	<40 copies per ml: n = 3	<40 copies per ml: n = 6
	≥40 copies per ml: n = 6	≥40 copies per ml: n = 4	≥40 copies per ml: n = 10
	unknown: n = 2		unknown: n = 2
Duration on LPV/r prior to tuberculosis treatment	4 (2–25) months	11 (9–31) months	10 (4–32) months
Median (IQR)	(n = 3)	(n = 5)	(n = 8)
Duration on tuberculosis treatment prior to LPV/r initiation	2 (1–2) months	1 month	1.5 (1–2) months
Median (IQR)^3^	(n = 7)	(n = 2)	(n = 9)
Month of tuberculosis treatment when enrolled, by patient	Patient 1 = 3	Patient 8 = 3	Median (IQR) = 4 (2–5)
	Patient 2 = 7	Patient 9 = 3	
	Patient 3 = 4	Patient 10 = 1	
	Patient 4 = 2	Patient 11 = 3	
	Patient 5 = 4	Patient 12 = 3	
	Patient 6 = 2	Patient 13 = 6	
	Patient 7 = 6	Patient 14 = 4	
	Median(IQR) = 4 (2–4.5)	Patient 15 = 4	
		Patient 16 = 4	
		Patient 17 = 5	
		Patient 18 = 2	
		Median (IQR) = 4 (2.5–5)	
Number of pharmacokinetic measurements during study period, by patient	Patient 1 = 3	Patient 8 = 3	Median (IQR) = 3 (2–4)
	Patient 2 = 2	Patient 9 = 3	
	Patient 3 = 1	Patient 10 = 1	
	Patient 4 = 5	Patient 11 = 3	
	Patient 5 = 1	Patient 12 = 3	
	Patient 6 = 5	Patient 13 = 6	
	Patient 7 = 3	Patient 14 = 4	
	Median (IQR) = 3 (2–4)	Patient 15 = 4	
		Patient 16 = 4	
		Patient 17 = 5	
		Patient 18 = 2	
		Median (IQR) = 3 (3–4)	

1 CD4-counts were collected from the clinical record. We recorded the last CD4-count prior to study enrolment.

2 Viral load measurements were collected from the clinical record. We recorded the last viral load prior to study enrolment that was done within 6 months of tuberculosis diagnosis and treatment.

3 One patient was started on LPV/r-based ART and tuberculosis treatment on the same day in the double dose LPV/r group.

**Figure 2:**
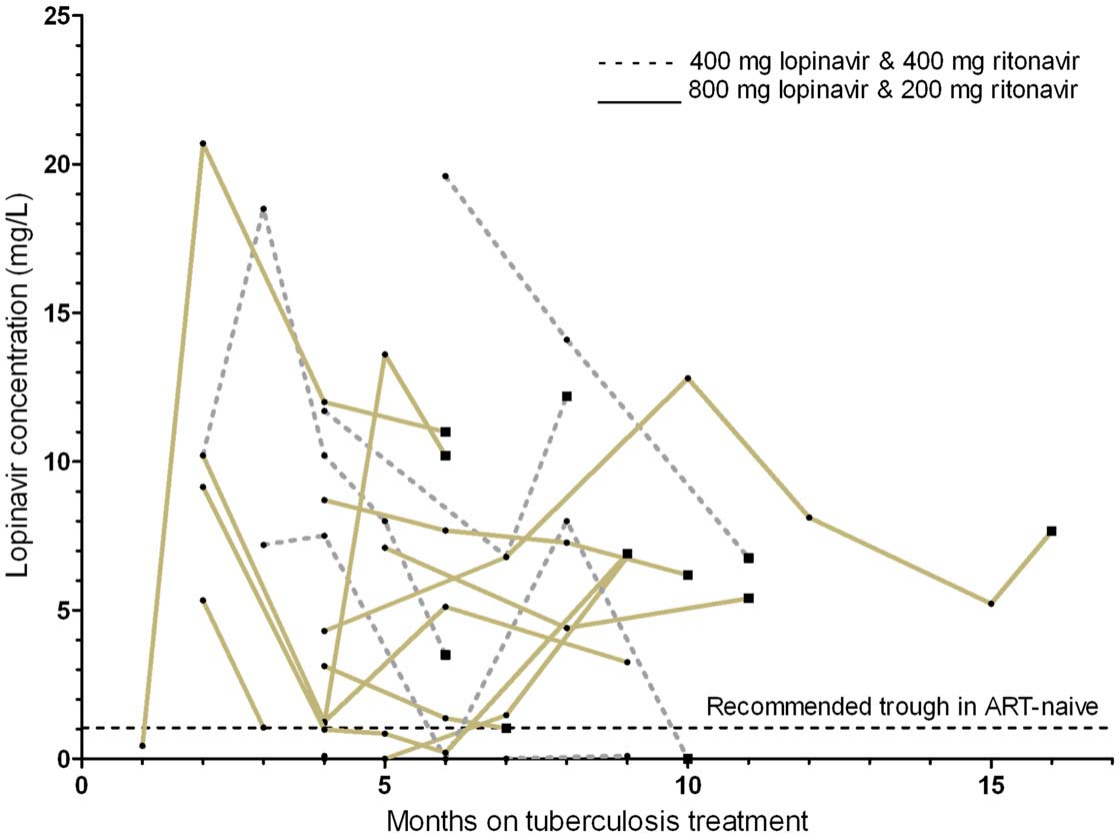
Lopinavir concentrations of individual patients during the study period. The circles indicate lopinavir concentrations measured while patients were receiving tuberculosis treatment, while the squares indicate lopinavir concentrations once tuberculosis treatment has been completed.

Lopinavir pre-dose concentrations were measured on a total of 58 study visits, of which 47 were while patients were receiving tuberculosis treatment. Lopinavir concentrations were measured 10 h22–18 h20 hours after the evening dose. During tuberculosis treatment, the median (IQR) LPV pre-dose concentration across all study visits was 6.8 (1.1–9.2) mg/L, and 36/47 (77%) measures were above the recommended trough concentration of 1 mg/L (Figure 2) [12], [13]. The median lopinavir pre-dose concentration was 6.2 mg/L in patients receiving additional ritonavir, 5.8 mg/L in patients receiving double dose lopinavir, and 6.8 mg/L in patients after tuberculosis therapy. We reduced the dose of LPV/r from 4 to 3 tablets 12 hourly in a patient who developed intolerable diarrhoea. During tuberculosis treatment 36/47 (77%) LPV pre-dose concentrations were above the recommended trough concentration of 1 mg/L [12], [13]. Single lopinavir pre-dose concentrations <1 mg/L, with all others being >1 mg/mL without dose adjustments, were observed during tuberculosis treatment in 3 patients each in the additional ritonavir and double dose LPV/r groups. The intra-individual variability in lopinavir concentrations was most likely due to variability in adherence. One patient in the additional ritonavir group had 2 lopinavir pre-dose concentrations <1 mg/L. She had an unsuppressed viral load and a lopinavir pre-dose concentration <1 mg/L after she completed tuberculosis treatment and has since defaulted ART care. Ten of the 11 patients that were followed up until tuberculosis treatment completion had undetectable viral loads. The median (IQR) lopinavir pre-dose concentration after tuberculosis treatment was 6.8 (3.5–10.2) mg/L.

There were no adverse events of grade 3 or 4 toxicity. Ten patients developed adverse events, 5 in each dosing group: 8 patients developed asymptomatic grade 1 or 2 transaminase elevation, 1 patient defaulted additional ritonavir due to nausea and 1 patient in the double dose group developed diarrhea requiring dose reduction as noted above. The patient who received a dose reduction had a suppressed viral load after tuberculosis treatment completion. No transaminitis was recorded in the patient who was HBsAg positive.

## Discussion

We found that lopinavir pre-dose concentrations were adequate in patients on rifampicin-based tuberculosis treatment. Most patients had lopinavir pre-dose concentrations >1 mg/L with adjusted LPV/r doses, confirming that adequate lopinavir concentrations can be obtained in the presence of rifampicin-based tuberculosis treatment by doubling the dose of LPV/r or adding additional ritonavir. The isolated subtherapeutic measures were most likely due to poor adherence.

The low rate of hepatotoxicity we observed is consistent with our previous study of HIV-infected patients established on LPV/r who were given rifampicin [4]. However, our previous findings were limited because we did not study patients with tuberculosis, other antitubercular drugs were not given, and follow up was brief. The inhibitory effect of isoniazid on drug metabolizing enzymes [8], [9] in patients on combination tuberculosis treatment may attenuate the inducing effect of rifampicin, thus potentially resulting in higher lopinavir concentrations and altering the generation of reactive protease inhibitor metabolites which may predispose to hepatotoxicity [5]–[7]. In our cohort no hepatotoxicity severe enough to warrant treatment discontinuation occurred, but larger prospective studies need to confirm this. Proportionally more patients in the additional ritonavir group developed adverse events compared to the adjusted LPV/r group (5/7 compared to 5/11), but our study was not designed to detect adverse event differences between the dosing approaches.

Data in patients receiving co-treatment with LPV/r and rifampicin-based tuberculosis treatment is sparse as a limited number of patients are currently receiving second line treatment. L'homme et al retrospectively described their clinical experience of combining LPV/r and rifampicin in 5 patients who received the recommended increased dose of LPV/r, 2 of whom discontinued treatment within 4 weeks due to acute adverse events [14] A paediatric study found no significant difference in the proportions of children with grade 3/4 transaminitis receiving tuberculosis treatment and adjusted doses of LPV/r compared with children on LPV/r alone [13].

Our study findings have several limitations. First, as most of our study patients were enrolled once they were established on tuberculosis treatment for several months, we may have under-estimated toxicity by failing to identify patients who developed early adverse events. Second, our attrition rate was relatively high. These two limitations could have resulted in an under-estimation of the toxicity of adjusted doses of LPV/r with rifampicin-based antitubercular therapy. Third, the low lopinavir concentrations we observed were likely due to missed doses that we did not detect as we used self-report as an adherence measure, which is known to be very insensitive. Fourth, due to the nature of the study, the time post-dose of lopinavir concentrations varied between patients (10 h22–18 h20 hours after the evening dose) increasing the variability around our pre-dose lopinavir concentration estimates. Lastly, our conclusions are limited by our small sample size.

The additional ritonavir dosing approach is complicated by the increased pill burden and low temperature storage instructions. Furthermore in our study and in a healthy volunteer study there was a trend towards more toxicity in the additional ritonavir dosing group, although neither study was adequately powered to detect a difference in toxicity [3]. Nevertheless it is well established that high doses of ritonavir are poorly tolerated. We therefore suggest that double dose LPV/r be used in adequately powered studies of tuberculosis patients in order to assess safety and efficacy. Until further data are available it would be prudent to monitor transaminases regularly during co-treatment. Our approach has been to increase the LPV/r dose by 50% after a week of anti-tuberculosis therapy and to double the dose after a further week.

In conclusion, we found that once patients are established on treatment adjusted doses of LPV/r co-administered with rifampicin-based tuberculosis, treatment was tolerated and lopinavir pre-dose concentrations were adequate. Further research is required to better describe safety during the early period when tuberculosis treatment is initiated in patients on LPV/r-based ART.
